# Concomitant Use of Proton Pump Inhibitors and Palbociclib Among Patients With Breast Cancer

**DOI:** 10.1001/jamanetworkopen.2023.24852

**Published:** 2023-07-21

**Authors:** Ju-Eun Lee, Sun-Hong Kwon, Swan Kwon, Hye-In Jung, Jin Hyun Nam, Eui-Kyung Lee

**Affiliations:** 1 School of Pharmacy, Sungkyunkwan University, Gyeonggi-do, Republic of Korea; 2 Division of Big Data Science, Korea University Sejong Campus, Sejong, Republic of Korea

## Abstract

**Importance:**

Proton pump inhibitors (PPIs) are commonly used drugs to relieve gastrointestinal tract symptoms, but their acid-inhibitory action negatively affects the bioavailability and clinical outcomes of orally administered concomitant drugs.

**Objective:**

To identify the clinical outcomes of patients with advanced breast cancer who concomitantly use PPIs and palbociclib.

**Design, Setting, and Participants:**

This retrospective cohort study used nationwide claims data between November 1, 2016, and July 31, 2021, in South Korea. Patients with breast cancer receiving palbociclib between November 1, 2017, and July 31, 2020, were identified. Patients whose prescriptions for palbociclib and PPI overlapped by at least 33% were classified into a concomitant PPI group. Patients who never received PPI during the palbociclib treatment period were classified into a nonconcomitant PPI group. Patients were selected through 1:3 propensity score matching for analyses.

**Exposures:**

Concomitant use of PPIs with palbociclib.

**Main Outcomes and Measures:**

Time to progression and death. These outcomes were presented as progression-free survival (PFS) and overall survival (OS) and were analyzed using the Kaplan-Meier method and log-rank test. Cox proportional hazards regression was used to estimate the hazard ratio (HR) of concomitant PPI use associated with clinical PFS and/or OS.

**Results:**

A total of 344 women were included in the concomitant PPI group and 966 in the nonconcomitant PPI group. Among 1310 patients identified after matching, 1108 (84.6%) were older than 50 years; 1111 (84.8%) were treated with letrozole and anastrozole (endocrine sensitive); and 199 (15.2%) were treated with fulvestrant (endocrine resistant). The median clinical PFS in the concomitant PPI group was shorter than that of the nonconcomitant PPI group (25.3 [95% CI, 19.6-33.0] vs 39.8 [95% CI, 34.9 to not applicable] months; *P* < .001), and the HR was 1.76 (95% CI, 1.46-2.13). Concomitant use of PPI was also associated with shorter OS (HR, 2.71 [95% CI, 2.07-3.53]). Both clinical PFS and OS in the concomitant PPI group were consistently poor in patients receiving endocrine-sensitive and endocrine-resistant treatment.

**Conclusions and Relevance:**

These findings suggest that concomitant use of PPIs with palbociclib may hinder the complete therapeutic benefits of palbociclib in patients with breast cancer.

## Key Points

**Question:**

Is concomitant use of palbociclib with a proton pump inhibitor (PPI) associated with a higher risk of progression among patients with advanced or metastatic breast cancer?

**Findings:**

In this cohort study of 1310 South Korean women with breast cancer identified using nationwide claims data, progression-free survival and overall survival in the concomitant PPI group were shorter than those in the nonconcomitant PPI group.

**Meaning:**

These findings suggest that taking PPIs with palbociclib may interrupt the full therapeutic benefits of palbociclib.

## Teaser

This cohort study identifies the clinical outcomes of patients with hormone receptor–positive and *ERBB2*-negative advanced or metastatic breast cancer who concomitantly use proton pump inhibitors and palbociclib.

## Introduction

Most patients with cancer use proton pump inhibitors (PPIs) to mitigate anticancer drug–related gastrointestinal symptoms, such as gastroesophageal reflux disease.^1^ Proton pump inhibitors irreversibly bind to and inhibit the hydrogen-potassium adenosine triphosphatase pump located on the luminal surface of the parietal cell membrane, reducing the secretion of gastric acid.^2,3^ However, acid suppression negatively affects the oral bioavailability, pharmacokinetics, and clinical effects of orally administered anticancer medications.^4,5^ For this reason, PPIs could be considered to have a high risk of drug-drug interaction with other anticancer drugs.

Cyclin-dependent kinase 4 and 6 (CDK4/6) inhibitors for hormone receptor (HR)–positive and *ERBB2* (previously known as *HER2*)-negative advanced or metastatic breast cancer have changed the landscape of treatment for breast oncology.^6^ Palbociclib, an oral small molecular inhibitor of CDK4/6, has been recommended in combination with aromatase inhibitors or fulvestrant as a treatment for patients with HR-positive and *ERBB2*-negative advanced or metastatic breast cancer.^7^ Palbociclib is a weak base medication whose absorption and blood concentration can also be decreased by coadministering a PPI, resulting in poor clinical efficacy. The first US Food and Drug Administration approval of palbociclib as an oral capsule noted that concurrent PPI use reduced exposures of palbociclib based on 62% of the area under the plasma concentration time curve from time 0 to infinity and 80% of the maximum plasma palbociclib concentration. With regard to these associations, several related prior studies^8,9,10,11^ have reported that a combination of a PPI and palbociclib, a CDK4/6 inhibitor, changes the therapeutic effect (with shorter progression-free survival [PFS]) of patients with HR-positive, *ERBB2*-negative advanced and metastatic breast cancer.

To our knowledge, insufficient data and small sample size have limited retrospective studies reporting drug-drug interactions between PPIs and palbociclib. In addition, some studies^10,11^ have shown no statistically significant differences and have reported the small number of patients as a limitation. Therefore, studies targeting more patients are needed to investigate the effects of concurrent PPI and palbociclib administration. In this study, we aimed to identify the clinical outcomes of patients with HR-positive and *ERBB2*-negative advanced or metastatic breast cancer who concomitantly use PPIs and palbociclib, based on South Korean claims data.

## Methods

### Data Source and Ethics

For this cohort study, we obtained nationwide claims data from the Health Insurance Review and Assessment Service in South Korea. As health insurance in South Korea is provided as a single-payer system, the database contains information regarding medical treatment, medicines, and medical resources for a total population of 50 million people.^12^ We collected demographic characteristics such as age and sex and medical information such as disease diagnosis and medical drug use from the database to identify eligible patients. We used data from November 1, 2016, to July 31, 2021 (the study period). During the study period, we did not observe the use of palbociclib as a tablet, but a capsule. This is because in South Korea, the palbociclib tablet was approved only in February 2022. This study was approved by the institutional review board of Sungkyunkwan University, which did not require informed consent for the use of retrospective deidentifed data. The study followed the Strengthening the Reporting of Observational studies in Epidemiology (STROBE) reporting guideline.

### Study Design and Patient Selection

In this retrospective cohort study, we assessed the clinical outcomes of patients with breast cancer and concomitant use of PPIs with palbociclib. The patient identification period was from November 1, 2017—the reimbursement date for palbociclib—to July 31, 2020. In the study period, the palbociclib taken by all patients was in capsule formulation. We identified women with breast cancer based on the presence of at least 2 claims with code C50 from the *International Statistical Classification of Diseases and Related Health Problems, Tenth Revision* (*ICD-10*), during the study period. They were required to use palbociclib for at least 1 cycle, during which the drug was administered continuously for 21 days. We excluded patients who used drugs targeting *ERBB2* such as trastuzumab, trastuzumab emtansine, pertuzumab, and lapatinib ditosylate during the study period. A detailed patient selection flow is shown in eFigure 1 in Supplement 1. Cohort entry was defined as the date of the first palbociclib prescription (eFigure 2 in Supplement 1). The date of first PPI use was defined as the index date. Patients were assessed from the index date for outcomes.

Baseline characteristics of the patients were assessed by index date (age, menopause, and treatment combination) or from 1 year prior to the index date (metastases, Charlson Comorbidity Index score, prior chemotherapy, and prior endocrine therapy) (eFigure 2 in Supplement 1). Palbociclib is approved in combination with an aromatase inhibitor as a first-line endocrine therapy and in combination with fulvestrant for disease progression after endocrine therapy. Both regimens are applicable to postmenopausal patients; however, they are only applicable for reimbursement to premenopausal women when both regimens are administered with ovarian function suppressants. As the claims data could not confirm the menopausal status of the patient, this was classified based on the use of ovarian function suppressants (leuprorelin or goserelin acetate).

### Exposure

The concomitant PPI group was defined as those who were coadministered PPI for more than one-third of the palbociclib treatment duration, while those who did not take any PPI in this period were classified as the nonconcomitant PPI group. We assumed patient medication adherence based on the date of prescription and the number of days supplied for each prescription. The PPIs included in this study were dexlansoprazole (Anatomical Therapeutic Chemical [ACT] code A02BC06), esomeprazole magnesium (ACT code A02BC05), ilaprazole (ACT code A02BC), lansoprazole (ACT code A02BC03), omeprazole (ACT code A02BC01), pantoprazole sodium (ACT code A02BC02), and rabeprazole sodium (ACT code A02BC04).

We performed 1:3 propensity score matching by matching 1 patient from the concomitant PPI group with 3 patients from the nonconcomitant PPI group to balance the characteristics of both groups. Using a logistic regression model, we estimated propensity scores of patients, including age, menopause, treatment combination, Charlson Comorbidity Index, whether prior chemotherapy or endocrine therapy was administered, and whether metastases had occurred. We additionally conducted analyses in 1:5 matched patients to see the robustness of results with reducing the loss of patients.

Patients were followed up after the index date to avoid immortal time bias,^13^ including events that occurred before the first prescription of PPI from the cohort entry. The index date for non-PPI users was specified as the interval between their cohort entry date and the index date of matched PPI users. Patients were excluded if the index date assigned by the matched PPI users was after the last palbociclib prescription date (eFigure 3 in Supplement 1).

### Outcomes

The outcomes measured were clinical PFS and overall survival (OS). As a measure of clinical PFS, we used the time to next treatment (TTNT), which estimates the time from the index date to the start of the next line of treatment or death. Time to next treatment has been used as a proxy for disease progression in patients since it is impossible to determine whether the patient’s disease has progressed in the claims data. As one might conclude that a patient commenced a new therapy because they experienced progression during their previous therapy, TTNT has been used as a proxy for time to progression in several analyses.^14^ By taking into account the progression of medication tolerability and patient adherence over time, TTNT provides a more accurate depiction of patient treatment experiences than traditional disease-related end points.^15^ It is also validated that TTNT may serve as a significant interim objective for the OS of patients with metastatic breast cancer.^16^ Therefore, TTNT was measured to represent clinical PFS in the study. Chemotherapy, immunotherapy, or endocrine therapy, such as tamoxifen, exemestane, and megestrol acetate, were defined as the next line of treatment if they were prescribed after the index date.

We estimated OS from the index date to last follow-up or death. We identified death if any one of *ICD-10* codes I461, R96, R98, or R99 was recorded or when the result of treatment was coded as death.

### Statistical Analysis

#### Main Analysis

Baseline characteristics are presented as mean (SD) for continuous variables and frequency for all categorical variables. A standardized mean difference was obtained to confirm whether the characteristics of the 2 groups were well balanced. In addition, the statistical significance of the difference between the concomitant and nonconcomitant groups was determined using unpaired *t* tests and χ^2^ tests, with a 2-sided *P* < .05 indicating statistical significance. We used the Kaplan-Meier method to generate survival curves and estimate patient median survival time. The log-rank test was used to compare the 2 groups. Hazard ratios (HRs) were determined using the Cox proportional hazard model, adjusting for other covariates. All statistical analyses were conducted using SAS Enterprise Guide, version 6.1 (SAS Institute Inc) and R, version 3.5 (R Project for Statistical Computing).

#### Subgroup and Sensitivity Analysis

We conducted subgroup analysis to compare the associations of concomitant PPI use among patients with different disease progression status. Since palbociclib is reimbursed in combination with an aromatase inhibitor as an initial endocrine-based therapy and with fulvestrant in patients with disease progression following initial endocrine therapy, disease in patients treated with fulvestrant is more advanced. To identify the outcomes of PPI after excluding patient disease factors, we assessed outcomes by classifying the patient groups according to whether they were sensitive to palbociclib plus letrozole or anastrozole or resistant to endocrine therapy consisting of palbociclib plus fulvestrant.

In the sensitivity analysis, we redefined the definition of the concomitant PPI group by changing the concomitant period to 50%, 67% (two-thirds of the period), and 80%. Matching with the nonconcomitant PPI group was repeated whenever the coverage ratio defining the concomitant group was changed. This analysis demonstrated how the results could vary depending on assumptions of the operational definition of the concomitant PPI group. In addition, we conducted a landmark analysis in which only patients who had survived until the landmark time were analyzed from those time points.^17^ The landmarks included were 3, 6, and 12 months after the initiation of the PPI. The robustness of the results was confirmed through landmark analyses.

## Results

Table 1 presents the baseline demographic and clinical characteristics of the patients before and after propensity score matching. After matching the 1310 selected patients, 344 women were determined to be in the concomitant PPI group and 966 in the nonconcomitant PPI group (eFigure 1 in Supplement 1). The standardized mean differences for all patient characteristics, except for lung metastasis, were less than 0.1. Most of the patients (1108 [84.6%]) were older than 50 years and had attained menopause (1289 [98.4%]). Palbociclib was combined with nonsteroidal aromatase inhibitors such as anastrozole or letrozole in 1111 patients (84.8%). Most patients had not received chemotherapy (1298 [99.1%]) or endocrine therapy (1240 [94.7%]) prior to palbociclib. Half of the patients in both groups had metastases. Nearly one-third of patients in both groups (368 [28.1%]) developed bone metastasis, the most frequent site of metastasis in both groups.

**Table 1.** Baseline Characteristics Before and After 1:3 Propensity Score Matching^a^

Characteristic	Unmatched patients (n = 2352)				Matched patients (n = 1310)			
	Concomitant PPI group (n = 344)	Nonconcomitant PPI group (n = 2008)	*P* value^b^	SMD	Concomitant PPI group (n = 344)	Nonconcomitant PPI group (n = 966)	*P* value^b^	SMD
Age, y								
≤50	53 (15.4)	612 (30.5)	<.001	0.36	53 (15.4)	149 (15.4)	>.99	0.00
>50	291 (84.6)	1396 (69.5)			291 (84.6)	817 (84.6)		
Menopause								
Yes	338 (98.3)	1981 (98.7)	.62	−0.03	338 (98.3)	951 (98.4)	.80	−0.02
No	6 (1.7)	27 (1.3)			6 (1.7)	15 (1.6)		
Treatment combination								
Anastrozole or letrozole	292 (84.9)	1673 (83.3)	.53	−0.04	292 (84.9)	819 (84.8)	>.99	−0.00
Fulvestrant	52 (15.1)	335 (16.7)			52 (15.1)	147 (15.2)		
CCI score, mean (SD)^c^	5.40 (3.38)	4.72 (3.37)	.003	0.20	5.40 (3.38)	5.23 (3.36)	.44	0.05
Prior chemotherapy								
Yes	4 (1.2)	39 (1.9)	.39	−0.06	4 (1.2)	8 (0.8)	.53	0.03
No	340 (98.8)	1969 (98.1)			340 (98.8)	958 (99.2)		
Prior endocrine therapy								
Yes	20 (5.8)	116 (5.7)	>.99	0.00	20 (5.8)	50 (5.2)	.68	0.03
No	324 (94.2)	1892 (94.2)			324 (94.2)	916 (94.8)		
Any metastasis	174 (50.6)	984 (49.0)	.60	0.03	174 (50.6)	517 (53.5)	.38	−0.06
Bone^d^	104 (30.2)	522 (26.0)	.11	0.09	104 (30.2)	264 (27.3)	.33	0.06
Lung^d^	44 (12.8)	301 (15.0)	.32	−0.06	44 (12.8)	174 (18.0)	.03	−0.15
Brain^d^	9 (2.6)	39 (1.9)	.41	0.05	9 (2.6)	15 (1.6)	.24	0.07
Liver^d^	15 (4.4)	104 (5.2)	.60	−0.04	15 (4.4)	49 (5.1)	.66	−0.03
Other sites^d^	23 (6.7)	114 (5.7)	.46	0.04	23 (6.7)	67 (6.9)	>.99	−0.01

Abbreviations: CCI, Charlson Comorbidity Index; PPI, proton pump inhibitor; SMD, standardized mean difference.

^a^ Unless otherwise indicated, data are expressed as No. (%) of patients. Percentages have been rounded and may not total 100.

^b^ The *P* value of the difference between the concomitant and nonconcomitant PPI groups was determined using unpaired *t* tests and χ^2^ tests.

^c^ Breast cancer was excluded when calculating CCI scores. Scores of 1 or 2 indicate mild; higher scores indicate greater severity.

^d^ Variables were not used to match the 2 groups.

**Figure 1.**
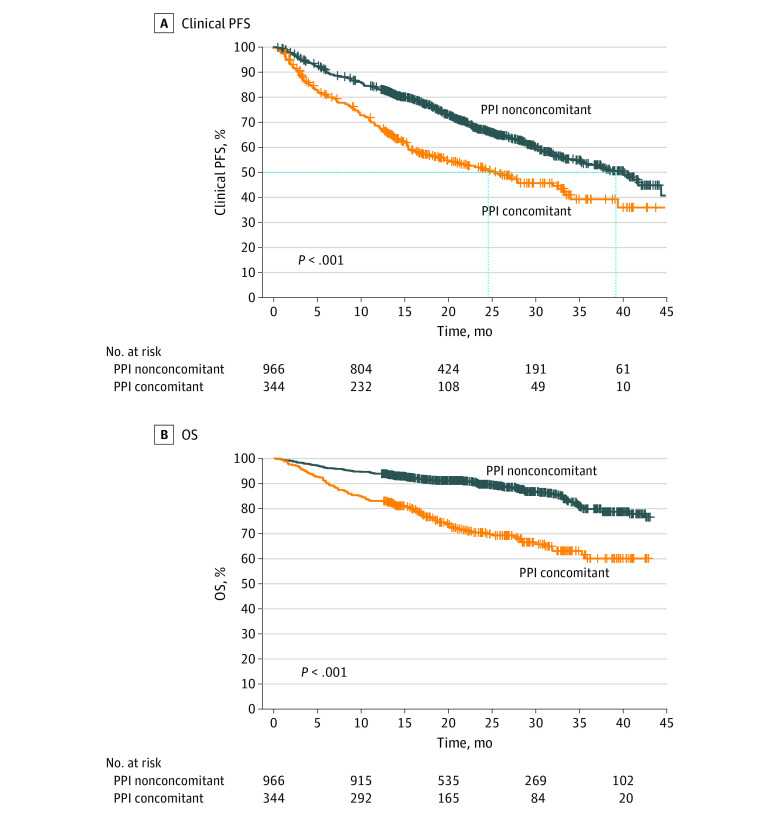
Progression-Free Survival (PFS) and Overall Survival (OS) Patients in the group receiving concomitant proton pump inhibitors (PPI concomitant group) were propensity score matched with the patients who were not receiving PPIs (nonconcomitant PPI group).

The median clinical PFS of the concomitant PPI group was 25.3 [95% CI, 19.6-33.0] months, significantly shorter compared with 39.8 [95% CI, 34.9 to not applicable] months for the nonconcomitant PPI group (*P* < .001) (Figure 1A). The OS was also shorter in the concomitant PPI group than in the nonconcomitant PPI group. The difference between the 2 groups was statistically significant (1-year OS, 83.1% vs 94.0%; 2-year OS, 69.5% vs 89.3%; *P* < .001) (Figure 1B), even though the median OS in both the groups was not reached. Absolute risk differences were 15 per 100 person-years in clinical PFS and 11 per 100 person-years in OS (eTable 1 in Supplement 1). When other variables were adjusted, the HR for concomitant PPI use associated with clinical PFS was 1.76 (95% CI, 1.46-2.13) and the HR for OS was 2.71 (95% CI, 2.07-3.53) (Table 2 and eTable 2 in Supplement 1).

**Table 2.** HRs of Clinical PFS and OS

Analysis	No. of patients with concomitant PPI/nonconcomitant PPI	HR (95% CI)	
		Clinical PFS	OS
Base case	344/966	1.76 (1.46-2.13)	2.71 (2.07-3.53)
Sensitivity, %^a^			
50	267/768	1.92 (1.58-2.34)	2.51 (1.87-3.36)
67	205/600	2.01 (1.60-2.52)	2.92 (2.09-4.08)
80	158/468	1.91 (1.48-2.47)	3.31 (2.23-4.90)
Landmark			
At 3 mo	122/330	7.19 (5.39-9.59)	2.87 (1.85-4.46)
At 6 mo	95/253	7.45 (5.32-10.45)	2.40 (1.37-4.18)
At 12 mo	56/156	7.53 (4.69-12.07)	3.82 (1.62-9.01)

Abbreviations: HR, hazard ratio; OS, overall survival; PFS, progression-free survival; PPI, proton pump inhibitor.

^a^ Sensitivity analysis according to the coverage ratio defining the PPI group.

**Figure 2.**
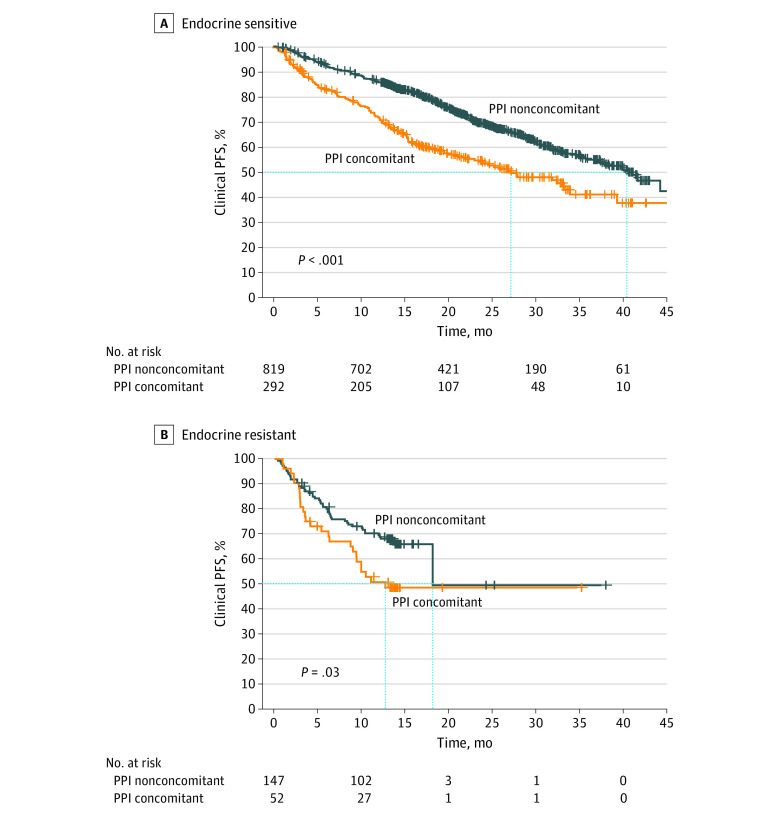
Subgroup Analysis of Progression-Free Survival (PFS) Patients receiving concomitant proton pump inhibitors (PPI concomitant group) and those who were not (nonconcomitant PPI group) were stratified between those who received endocrine-sensitive or endocrine-resistant therapy.

**Figure 3.**
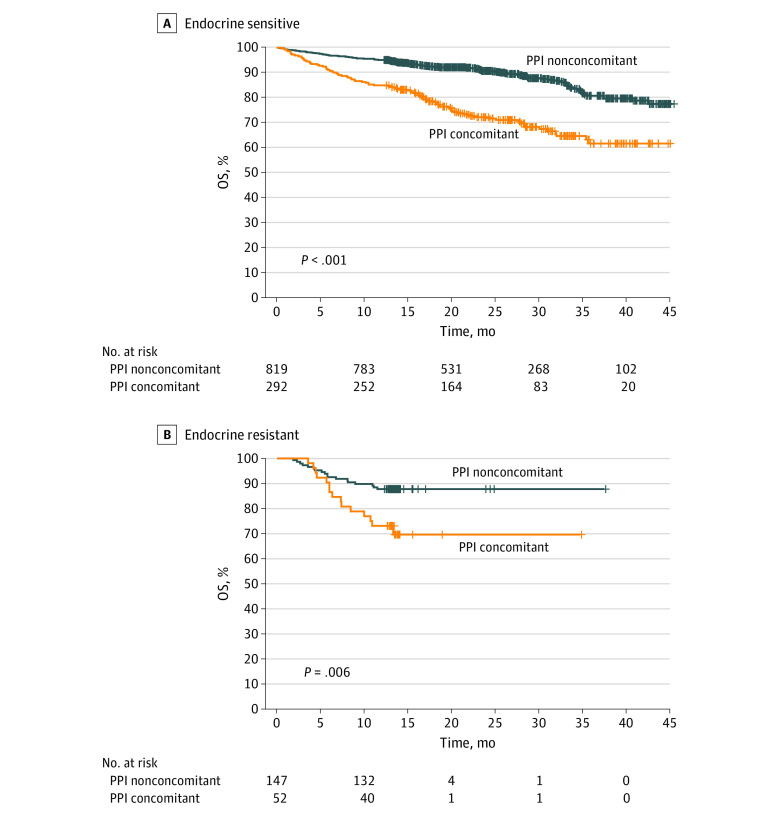
Subgroup Analysis of Overall Survival (OS) Patients receiving concomitant proton pump inhibitors (PPI concomitant group) and those who were not (nonconcomitant PPI group) were stratified between those who received endocrine-sensitive or endocrine-resistant therapy.

In the subgroup analysis (n = 344), 292 patients were classified as receiving endocrine-sensitive treatment and the remaining 52 patients were found to be receiving endocrine-resistant treatment. The median clinical PFS for the nonconcomitant PPI group of patients with endocrine-sensitive treatment was 40.4 [95% CI, 34.9 to not applicable] months, whereas the concomitant PPI group with endocrine-sensitive treatment had a median clinical PFS of 27.2 [95% CI, 20.6-34.0] months (*P* < .001). The HR for the endocrine-sensitive subgroup associated with clinical PFS was 1.75 (95% CI, 1.42-2.15) (Figure 2A). The endocrine-resistant subgroup also exhibited a substantial difference in clinical PFS between the 2 groups (adjusted HR, 1.82 [95% CI, 1.12-2.94]; *P* = .03) (Figure 2B). None of the groups reached a median OS. However, the difference between the 2 groups was statistically significant in all subgroups, and the HRs for taking PPIs with palbociclib associated with OS were 2.68 (95% CI, 2.01-3.58; *P* < .001) and 2.98 (95% CI, 1.49-5.96; *P* = .006) in the endocrine-sensitive and endocrine-resistant subgroups, respectively (Figure 3).

When we defined the concomitant PPI group by coverage ratios of 50%, 67%, and 80% as sensitivity analyses, the increased risk associated with clinical PFS and OS for taking PPI was robust in the sensitivity analysis (Table 2). A significant difference between the 2 groups was also consistently demonstrated. In patients who took a PPI for 80% or more of the duration of palbociclib treatment, concomitant PPI use increased the HR for clinical PFS to 1.91 (95% CI, 1.48-2.47) and for OS to 1.31 (95% CI, 2.23-4.90). The trend was also observed in the cohorts constructed with 3-, 6-, and 12-month landmarks (Table 2). In 1:5 matching that was performed to secure a somewhat larger number of patients, concomitant PPI use was associated with an increased risk to clinical PFS and OS (eTables 3-4 and eFigures 4-6 in Supplement 1).

## Discussion

Several studies^8,9,10,11^ have demonstrated that using palbociclib concurrently with PPIs leads to inferior clinical results for patients, particularly with regard to PFS. We attempted to obtain a greater number of patients for analysis by using nationwide claims data to address the issue of limited sample sizes in previous studies. As an indicator of the results, we attempted to determine when the disease actually progressed and the next treatment was started. Additionally, we analyzed OS, which has been relatively unexplored in previous research.

We found that in patients with advanced or metastatic breast cancer, taking PPIs concomitantly with palbociclib was associated with considerably reduced clinical PFS and OS compared with those who did not take PPIs during the entire palbociclib treatment period, supporting the findings of earlier studies.^8,9,10,11^ In this study, the clinical PFS of patients with breast cancer receiving palbociclib and PPIs in combination was approximately 15 months shorter. After adjustment for other risk factors, the HR associated with taking PPI concomitantly appeared to be 1.76 for clinical PFS. Del Re et al^8^ reported that concomitant PPI increases progression by an HR of 2.77 (95% CI, 1.62-4.75), and Eser et al (95% CI, 2.67-23.05)^9^ reported an HR of 7.85. Compared with earlier research, the HR in our study was more conservative, but the results that concomitant use of PPIs was associated with the patient clinical outcomes were mostly consistent. While the early separation of PFS and OS were observed in our study, the trends were similar with PFS from the previous studies.^8,9,10^

Reis et al^10^ (2022) concluded that survival outcomes were not statistically significantly different between the 2 groups. However, the literature also implies that if a larger number of patients are followed up for a longer period of time, potential differences may become more apparent. From the previous literature, the nonconcomitant PPI group was as few as 40 patients, of whom fewer than 10 were included in each variable for subgroup analysis, making it difficult to determine whether the variables were significantly adjusted. Our study, which analyzed a larger number of patients, identified a statistically significant difference in survival outcomes (HR, 2.71). Results from landmark and sensitivity analyses also demonstrated conclusive evidence that the combination of PPI with palbociclib was associated with a higher risk of disease progression and death. We confirmed that concomitant PPI use was associated with an increase in the risk to clinical PFS and OS by performing subgroup analysis and classifying patients according to the combined treatment. Even when palbociclib was administered with fulvestrant, it appeared to significantly increase the risk of disease progression and death. This was an expected result because the treatment lines of the patients using palbociclib were different.

Proton pump inhibitors alter the gastrointestinal environment by decreasing gastric acid secretion, consequently influencing drug absorption.^18^ Based on the research that reported that patient clinical outcomes deteriorate when oral weakly basic anticancer medications such as tyrosine kinase inhibitors are used in combination with PPIs,^19,20,21^ it appears that complete absorption of palbociclib has been achieved, so it has not shown sufficient therapeutic performance. Even though the type of cancer studied was different from ours, this association was also confirmed from the previous studies.^2,20^ Ha et al^2^ reported a significant difference in PFS and OS between PPI and non-PPI groups in patients with advanced or metastatic renal cell cancer (40.9 vs 62.4 weeks; *P* = .02). Eser at al^9^ concluded that increasing gastric pH immediately induced by PPIs may occur through lowering palbociclib plasma concentrations, which affects treatment efficacy and results in shorter PFS. Likewise, it seems that the increase in gastric pH by PPI reduces the absorption and efficacy of weakly basic drugs such as palbociclib, which have pH-dependent solubility.

Similarly, we wondered whether PPI administration had similar associations with clinical outcomes for other weakly basic CDK4/6 inhibitors, such as ribociclib succinate and abemaciclib. Previous studies concerning palbociclib have also investigated the PPI effect of ribociclib, but the conclusions are inconsistent.^22^ Studies with larger numbers of patients are required to obtain reliable findings. As mentioned, the palbociclib taken by the patients in this study was limited to the capsule formulation. The relationship between concomitant PPI and palbociclib tablet formulation requires confirmation by further studies.

### Limitations

Our study has certain limitations. Owing to the characteristics of the claims data used in our analysis, we could not confirm whether the patients actually took the medication. However, patients who received multiple prescriptions for palbociclib together with a PPI for more than a certain period of time were selected, which means that patients who received concomitant PPI only once were not selected. Moreover, in claims data, it is difficult to measure how much PPI should be taken together for drug-drug interactions to interfere with the treatment outcome. We attempted to overcome this uncertainty to some extent by performing a sensitivity analysis by varying the definition of the PPI group.

## Conclusions

The findings of this cohort study suggest that concomitant PPI use with palbociclib in patients with breast cancer was associated with poorer treatment outcomes than among those not using PPIs. Physicians should be cautious when prescribing PPIs to patients who are receiving palbociclib and should inform patients about the risks of interaction to prevent inadequate prescription of PPI by others.

## Notes

Supplement 1. **eTable 1.** Incidence of Progression-Free Survival and Overall Survival **eTable 2.** Adjusted Hazard Ratio of Progression-Free Survival and Overall Survival **eTable 3.** Baseline Demographics and Clinical Characteristics After 1:5 Propensity Score Matching **eTable 4.** Adjusted Hazard Ratio of Progression-Free Survival and Overall Survival From 1:5 Matched Cohort **eFigure 1.** Patient Selection Flowchart **eFigure 2.** Study Design **eFigure 3.** Assigning Index Date of Patients With Nonconcomitant PPI **eFigure 4.** Progression-Free Survival and Overall Survival of Concomitant PPI Group and Nonconcomitant PPI Group From 1:5 Matched Cohort **eFigure 5.** Progression-Free Survival in Patients With Endocrine-Sensitive and Endocrine-Resistant Treatment From 1:5 Matched Cohort **eFigure 6.** Overall Survival in Patients With Endocrine-Sensitive and Endocrine-Resistant Treatment From 1:5 Matched Cohort

Supplement 2.
Data Sharing Statement
