# Supplementary material for: Concomitant Use of Proton Pump Inhibitors and Palbociclib Among Patients With Breast Cancer
Source: JAMA Netw Open. 2023 Jul 21;6(7):e2324852. doi: 10.1001/jamanetworkopen.2023.24852 (PMC10362477; doi:10.1001/jamanetworkopen.2023.24852)
Supplement: Supplement 1. — eTable 1. Incidence of Progression-Free Survival and Overall Survival eTable 2. Adjusted Hazard Ratio of Progression-Free Survival and Overall Survival eTable 3. Baseline Demographics and Clinical Characteristics After 1:5 Propensity Score Matching eTable 4. Adjusted Hazard Ratio of Progression-Free Survival and Overall Survival From 1:5 Matched Cohort eFigure 1. Patient Selection Flowchart eFigure 2. Study Design eFigure 3. Assigning Index Date of Patients With Nonconcomitant PPI eFigure 4. Progression-Free Survival and Overall Survival of Concomitant PPI Group and Nonconcomitant PPI Group From 1:5 Matched Cohort eFigure 5. Progression-Free Survival in Patients With Endocrine-Sensitive and Endocrine-Resistant Treatment From 1:5 Matched Cohort eFigure 6. Overall Survival in Patients With Endocrine-Sensitive and Endocrine-Resistant Treatment From 1:5 Matched Cohort [file jamanetwopen-e2324852-s001.pdf]

Supplementary Online Content

Lee JE, Kwon SH, Kwon S, Jung HI, Nam JH, Lee EK. Concomitant use of proton pump inhibitors with palbociclib among patients with breast cancer. *JAMA Netw Open*. 2023;6(7):e2324852. doi:10.1001/jamanetworkopen.2023.24852

eTable 1. Incidence of Progression-Free Survival and Overall Survival

eTable 2. Adjusted Hazard Ratio of Progression-Free Survival and Overall Survival

eTable 3. Baseline Demographics and Clinical Characteristics After 1:5 Propensity Score Matching

eTable 4. Adjusted Hazard Ratio of Progression-Free Survival and Overall Survival From 1:5 Matched Cohort

eFigure 1. Patient Selection Flowchart
eFigure 2. Study Design

eFigure 3. Assigning Index Date of Patients With Nonconcomitant PPI

eFigure 4. Progression-Free Survival and Overall Survival of Concomitant PPI Group and Nonconcomitant PPI Group From 1:5 Matched Cohort
eFigure 5. Progression-Free Survival in Patients With Endocrine-Sensitive and Endocrine-Resistant Treatment From 1:5 Matched Cohort
eFigure 6. Overall Survival in Patients With Endocrine-Sensitive and Endocrine-Resistant Treatment From 1:5 Matched Cohort

This supplemental material has been provided by the authors to give readers additional information about their work.

© 2023 Lee JE et al. *JAMA Network Open*.

eTable 1. Incidence of Progression-Free Survival and Overall Survival

	Concomitant PPI (n=344)			Non-concomitant PPI (n=966)			Absolute risk difference	Relative risk ratio
	Events	Person -years	Incidence rate	Events	Person -years	Incidence rate		
**Real-world progression-free survival**	160	459	0.35 (0.29-0.40)	319	1,595	0.20 (0.18-0.22)	0.15 (0.09-0.21)	1.74 (1.44-2.11)
**Overall survival**	102	604	0.17 (0.14-0.20)	118	1,881	0.06 (0.05-0.07)	0.11 (0.07-0.14)	2.69 (2.07-3.51)

PPI: proton pump inhibitor

eTable 2. Adjusted Hazard Ratio of Progression-Free Survival and Overall Survival

Covariates	Hazard ratio (95% CI)	
	**Real-world progression-free survival**	**Overall survival**
**Concomitant proton pump inhibitor**		
No	1.00	1.00
Yes	**1.76 (1.46–2.13)**	**2.71 (2.07–3.53)**
**Treatment combination**		
palbociclib+anastrozole/letrozole	1.00	1.00
palbociclib+fulvestrant	**2.23 (1.68–2.95)**	**2.18 (1.44–3.30)**
**Age**		
<50	1.00	1.00
≥50	0.91 (0.71–1.17)	1.38 (0.90–2.11)
**Charlson comorbidity index**	1.02 (0.99–1.04)	1.01 (0.97–1.06)
**Menopause**		
No	1.00	1.00
Yes	1.20 (0.55–2.65)	1.51 (0.36–6.39)
**Prior chemotherapy**		
No	1.00	1.00
Yes	0.83 (0.33–2.05)	0.68 (0.16–2.81)
**Prior endocrine therapy**		
No	1.00	1.00
Yes	0.80 (0.53–1.22)	0.71 (0.36–1.41)

Bold indicates *P*-value < 0.05.

© 2023 Lee JE et al. *JAMA Network Open*.

eTable 3. Baseline Demographics and Clinical Characteristics After 1:5 Propensity Score Matching

	Concomitant PPI group (N = 344)	Non-concomitant PPI group (N = 1,587)	*P*-value[1)]	SMD
**Age, n (%)**			0.0686	0.11
<50	53 (15.41)	313 (19.72)		
≥50	291 (84.59)	1274 (80.28)		
**Menopause, n (%)**			0.6183	-0.03
Yes	338 (98.26)	1565 (98.61)		
No	6 (1.74)	22 (1.39)		
**Treatment combination, n (%)**			0.4717	-0.05
anastrozole/letrozole	292 (84.88)	1319 (83.11)		
fulvestrant	52 (15.12)	268 (16.89)		
**CCI, mean (SD)**	5.40 (3.38)	4.80 (3.42)	0.0033	0.17
**Prior chemotherapy, n (%)**			1.00	-0.03
Yes	4 (1.16)	23 (1.45)		
No	340 (98.84)	1564 (98.55)		
**Prior endocrine therapy, n (%)**			1.00	-0.01
Yes	20 (5.81)	95 (5.99)		
No	24 (94.19)	492 (94.01)		
**Any metastasis, (%)**	174 (50.58)	813 (51.23)	0.5524	0.04
Bone[2)]	104 (30.23)	411 (25.90)	0.1065	0.10
Lung[2)]	44 (12.79)	242 (15.25)	0.2764	-0.07
Brain[2)]	9 (2.62)	25 (1.58)	0.1785	0.07
Liver[2)]	15 (4.36)	78 (4.91)	0.7813	-0.03
Other sites[2)]	23 (6.69)	90 (5.67)	0.4488	0.04

[1)] The *P*-value of the difference between the concomitant and non-concomitant groups was determined using Student's t-tests and Chi-squared tests.

[2)] Variables were not used to match two groups

PPI: proton pump inhibitor, SD: standard deviation, SMD: standardized mean difference

© 2023 Lee JE et al. *JAMA Network Open*.

eTable 4. Adjusted Hazard Ratio of Progression-Free Survival and Overall Survival From 1:5 Matched Cohort

Covariates	Hazard ratio (95% CI)	
	Real-world progression-free survival	Overall survival
**Concomitant proton pump inhibitor**		
No	1.00	1.00
Yes	1.87 (1.59–2.20)	2.76 (2.16–3.52)
**Treatment combination**		
palbociclib+anastrozole/letrozole	1.00	1.00
palbociclib+fulvestrant	1.94 (1.57–2.40)	2.13 (1.49–3.02)
**Age**		
<50	1.00	1.00
≥50	1.02 (0.85–1.24)	1.36 (0.96–1.92)
**Charlson comorbidity index**	1.01 (0.99–1.03)	1.01 (0.97–1.04)
**Menopause**		
No	1.00	1.00
Yes	1.36 (0.69–2.69)	1.89 (0.45–7.86)
**Prior chemotherapy**		
No	1.00	1.00
Yes	1.02 (0.58–1.79)	1.27 (0.55–2.93)
**Prior endocrine therapy**		
No	1.00	1.00
Yes	0.97 (0.71–1.31)	0.82 (0.47–1.41)

© 2023 Lee JE et al. *JAMA Network Open*.

eFigure 1. Patient Selection Flowchart

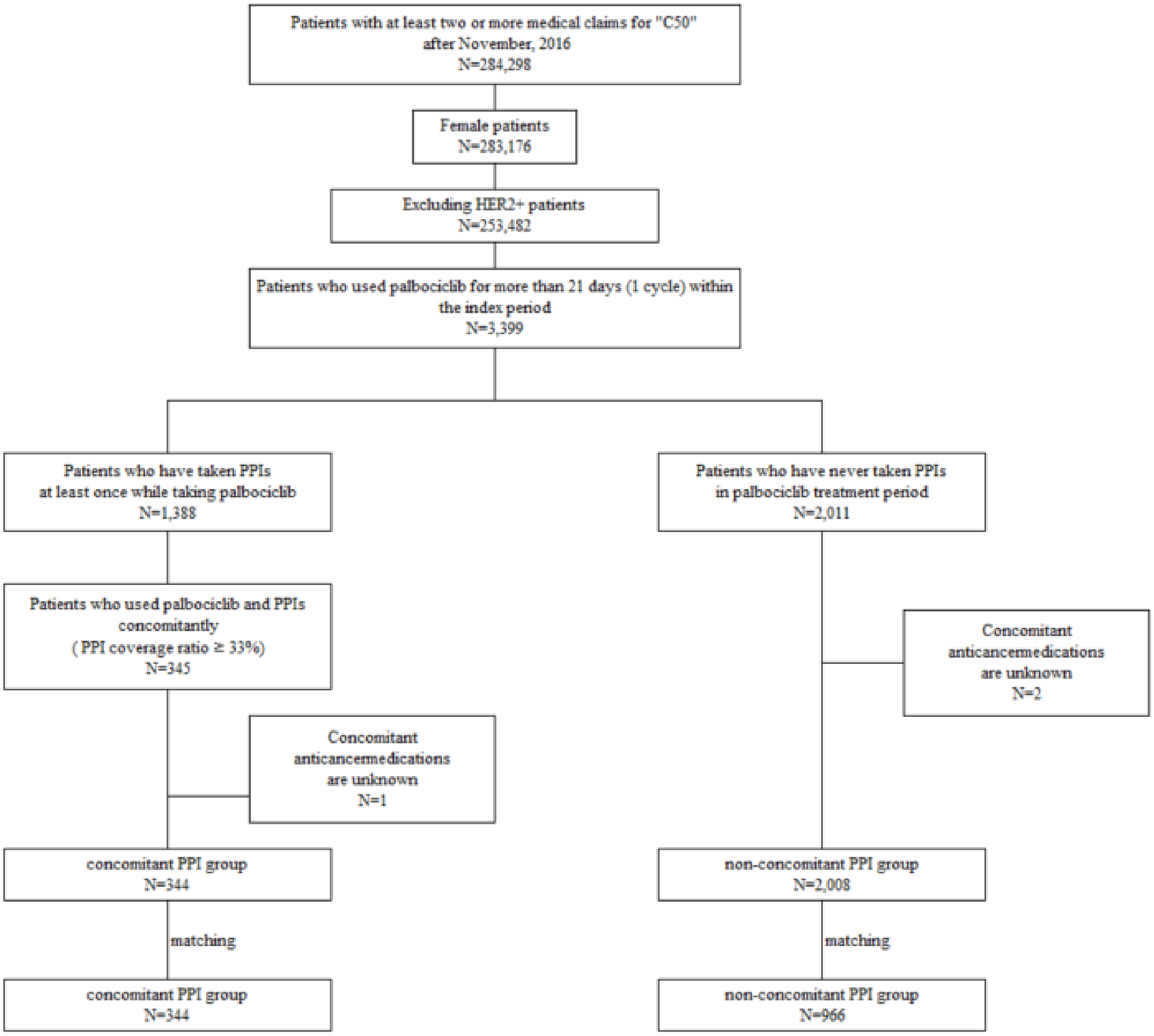

© 2023 Lee JE et al. *JAMA Network Open*.

eFigure 2. Study Design

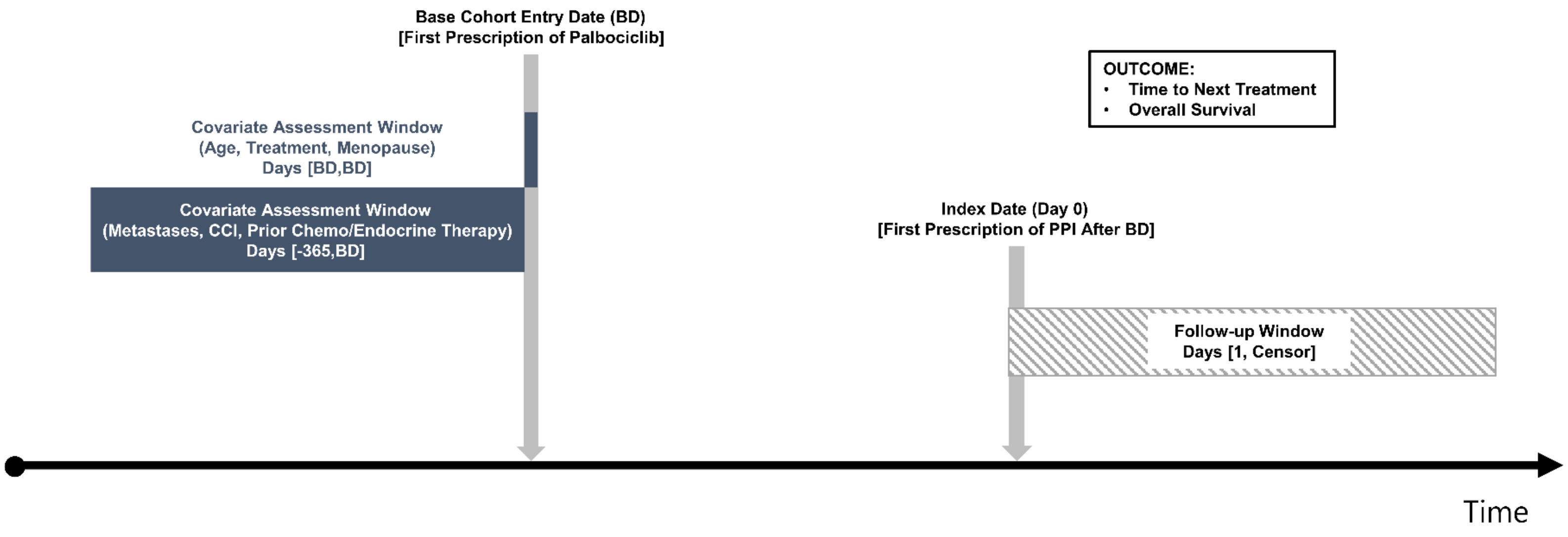

© 2023 Lee JE et al. *JAMA Network Open*.

eFigure 3. Assigning Index Date of Patients With Nonconcomitant PPI

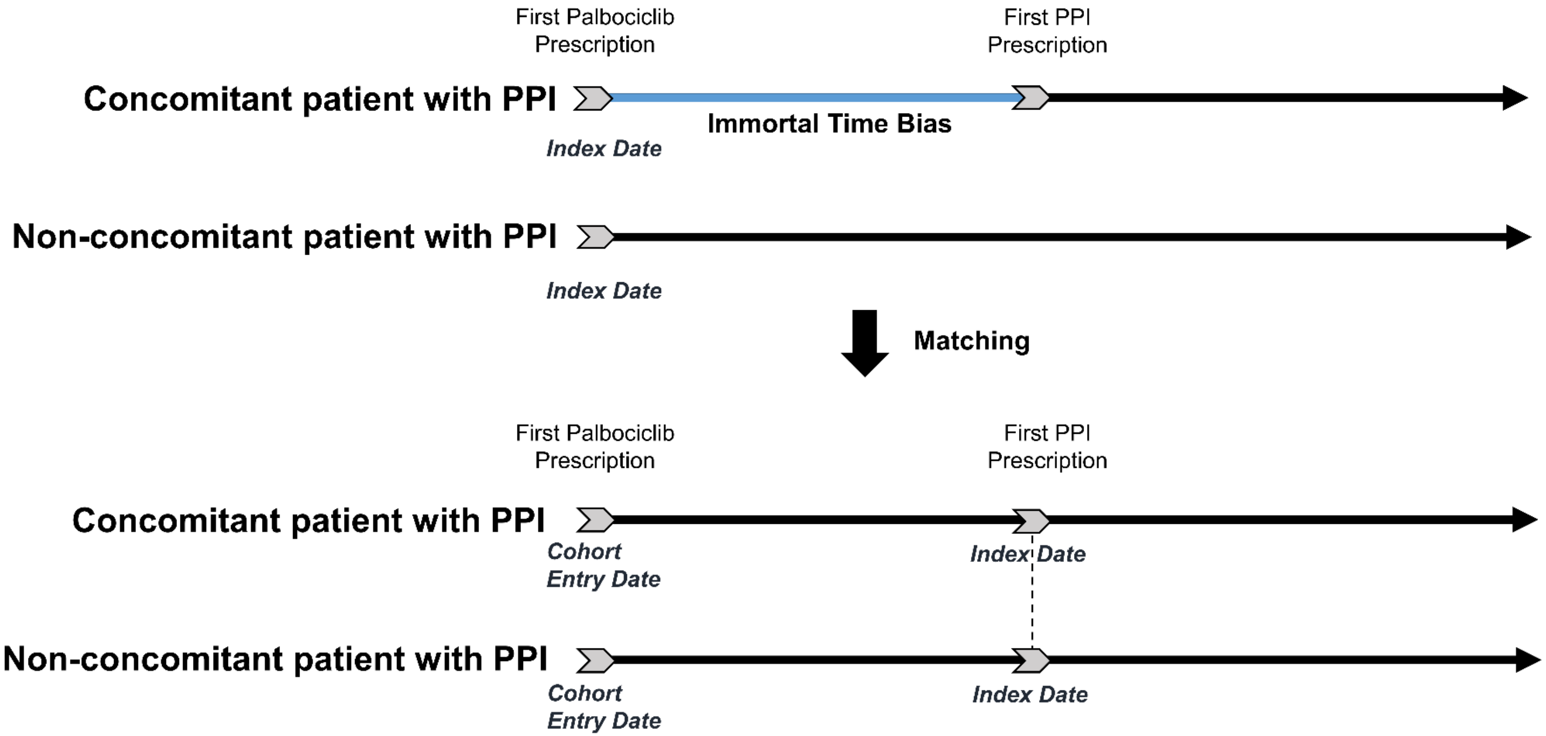

© 2023 Lee JE et al. *JAMA Network Open*.

eFigure 4. Progression-Free Survival and Overall Survival of Concomitant PPI Group and Nonconcomitant PPI Group From 1:5 Matched Cohort

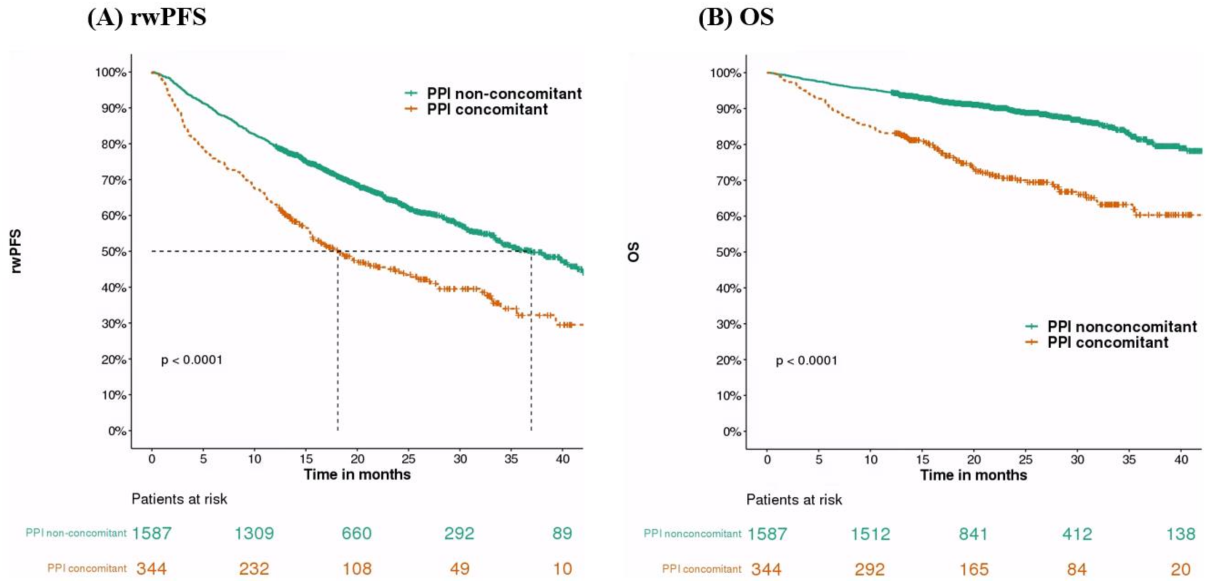

eFigure 5. Progression-Free Survival in Patients With Endocrine-Sensitive and Endocrine-Resistant Treatment From 1:5 Matched Cohort

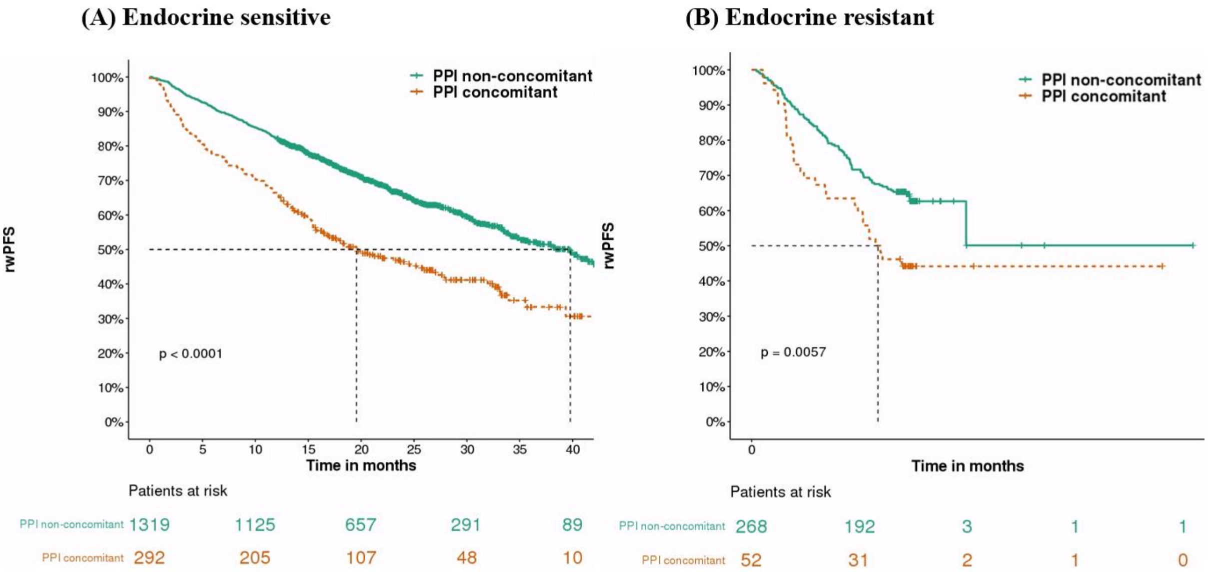

© 2023 Lee JE et al. *JAMA Network Open*.

eFigure 6. Overall Survival in Patients With Endocrine-Sensitive and Endocrine-Resistant Treatment From 1:5 Matched Cohort

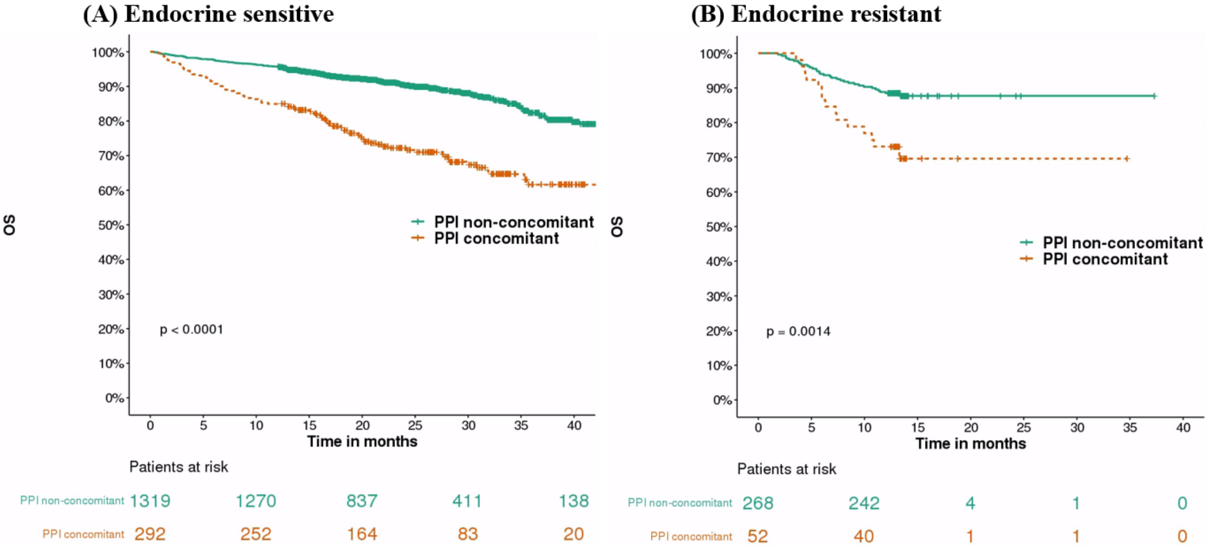

© 2023 Lee JE et al. *JAMA Network Open*.
