# Supplementary material for: Concomitant Use of Proton Pump Inhibitors and Palbociclib Among Patients With Breast Cancer
Source: JAMA Netw Open. 2023 Jul 21;6(7):e2324852. doi: 10.1001/jamanetworkopen.2023.24852 (PMC10362477; doi:10.1001/jamanetworkopen.2023.24852)
Supplement: Supplement 2. — Data Sharing Statement [file jamanetwopen-e2324852-s002.pdf]

Data Sharing Statement

Lee. Concomitant Use of Proton Pump Inhibitors and Palbociclib Among Patients With Breast Cancer. *JAMA Netw Open*. Published July 21, 2023.
doi:10.1001/jamanetworkopen.2023.24852

Data
Data available: No

Additional Information
Explanation for why data not available: The datasets generated in this study are potentially identifiable from the corresponding author on a reasonable request, but not publicly available due to ethical restrictions by the HIRA. The claims data may be available from HIRA with permission.
